# The hidden impact of smoking in thyroid eye disease: a link between selenium deficiency, autoimmunity, and retinal microvasculature

**DOI:** 10.20945/2359-4292-2026-0057

**Published:** 2026-06-08

**Authors:** Fatma Savur, Serkan Güler, Gülistan Oyur

**Affiliations:** 1 Istanbul Health Sciences University, Basaksehir Cam and Sakura City Hospital, Ophthalmology Department, Istanbul, Turkey

**Keywords:** Optical coherence tomography angiography, retinal microvasculature, selenium, smoking, thyroid-associated orbitopathy

## Abstract

**Objective:**

To investigate the impact of smoking on serum selenium levels, autoimmune activity, and macular microvascular density in patients with clinically inactive thyroid-associated orbitopathy (TAO), using optical coherence tomography angiography (OCTA).

**Subjects and methods:**

This retrospective cross-sectional study included 44 patients with inactive TAO (24 smokers, 20 non-smokers) and 32 ageand sex-matched healthy controls. All participants underwent ophthalmologic evaluation and laboratory testing for thyroid hormones, selenium, and thyroid-stimulating immunoglobulin (TSI). Macular vessel density was measured using spectral-domain OCTA at the superficial capillary plexus, deep capillary plexus, outer retinal layer, and choriocapillaris. Clinical activity score (CAS) was used to assess disease activity. The collected data were analyzed statistically.

**Results:**

Smokers with TAO exhibited significantly higher CAS (*p* = 0.016) and TSI levels (*p* = 0.027), and lower serum selenium concentrations (*p* = 0.042) compared to non-smokers. Central superficial and deep capillary plexus densities were significantly reduced in smokers versus healthy controls (*p* = 0.029 and *p* = 0.017, respectively). No significant differences were observed between smokers and non-smokers within the TAO group, or in the outer retinal layer and choriocapillaris layers among all groups.

**Conclusion:**

Smoking status was associated with lower selenium levels, higher TSI levels, and increased CAS. Furthermore, retinal microvascular attenuation detected by OCTA, even in the absence of clinical activity in TAO, may serve as a significant indicator of persistent vascular deterioration.

## INTRODUCTION

Thyroid-associated orbitopathy (TAO) is an autoimmune inflammatory disease that can occur in all thyroid conditions, but is most prevalent in patients with Graves’ disease (^1^). Although the orbital disease generally follows a mild course, it can lead to severe complications in 3-5% of cases. During the course of TAO, numerous individual risk factors have been identified, including phenotype, thyroid hormone dysfunction, and smoking (^2^-^4^). Multiple studies have investigated the relationship between TAO and smoking (^4^,^5^), and evidence has shown that smoking is the most significant modifiable risk factor for TAO, increasing the risk of severe disease forms and reducing treatment responsiveness in active TAO (^6^). *In vitro* studies have indicated that increased production of reactive oxygen species contributes to the pathogenesis of thyroid-related orbitopathy (^7^-^9^). Selenium is an antioxidant mineral required for selenocysteine synthesis (^10^); it is one of the agents that can potentially inhibit pathogenic mechanisms implicated in TAO. Deficiency has been shown to affect free radical formation in TAO, conversion of thyroxine (T4) to triiodothyronine (T3), and the autoimmune thyroid process (^11^). Nonetheless, there is limited research examining the effects of smoking on macular microvascular changes in inactive TAO (^12^).

Although previous studies have individually examined the roles of smoking, selenium, and retinal vascular changes in TAO, there remains a lack of comprehensive research exploring the interplay among these factors, particularly in patients with clinically inactive disease. Notably, the combined impact of smoking and selenium deficiency on both immunological activity and retinal microvascular integrity is largely unexplored. Given the critical role of oxidative stress and vascular compromise in TAO pathogenesis, it is essential to determine whether modifiable lifestyle factors such as smoking contribute to ongoing subclinical inflammation and ocular perfusion abnormalities, even during periods of clinical inactivity. Therefore, this study aims to comprehensively evaluate the effect of smoking on serum selenium levels, clinical activity score (CAS), thyroid autoantibodies, and macular microvascular parameters measured by optical coherence tomography angiography (OCTA) in patients with inactive TAO. By integrating biochemical, immunological, and imaging data, this study seeks to provide novel insights into the systemic and ocular consequences of smoking in TAO and to identify potential clinical targets for early risk stratification and therapeutic intervention.

## SUBJECTS AND METHODS

### Study population

This single-center, cross-sectional observational study was conducted at a tertiary eye care center. The retrospective study included the right eyes of 44 patients with inactive TAO (smoking group, n = 24) and 32 healthy participants (control group; ageand gender-matched). Informed consent was obtained from all participants, and the study was conducted in accordance with the principles of the Declaration of Helsinki. The protocol was reviewed and approved by the local ethics committee. All participants underwent comprehensive ophthalmologic examinations, including biomicroscopy, Hertel exophthalmometry, and indirect ophthalmoscopy. Demographic data, including age, gender, smoking status, and duration of TAO, were collected. Following thorough ophthalmological examination, CAS, thyroid-stimulating hormone (TSH), free thyroxine (fT4), free triiodothyronine (fT3), selenium (Se), and thyroid-stimulating immunoglobulin (TSI) levels were recorded for patients with TAO. The CAS system was used to assess disease activity. Patient and severity classification were performed using the European Group for Graves’ Ophthalmopathy criteria (^13^,^14^). Patients with CAS < 3 and no previous history of active TAO treatment were included.

Patients with active TAO or previous TAO treatment (e.g., orbital radiotherapy, systemic steroids, or other immunomodulators) were excluded. To prevent confounding effects on choroidal and retinal vasculature, participants with refractive errors greater than ±2 diopters, corneal opacities, cataracts, retinal vascular pathologies (e.g., diabetes, hypertension, systemic vasculitic diseases), and choroidal disease were excluded.

### Intervention and observation procedures

Vascular density measurements were performed using spectral-domain OCTA (DRI OCT Tritron, Topcon, Japan). The instrument acquires 6 × 6 mm macular volumetric scans with a sample density of 320 × 320 A-scans at a rate of 100,000 A-scans/second. The device employs an OCTA algorithm to generate angiograms (^15^). The algorithm uses blood flow as contrast and provides vessel density (VD) and flow field data based on the split-spectrum amplitude-correlation angiography algorithm. Participants fixated on the target following pupil dilation. Four measurements were obtained per participant, and images with a signal strength index >60 were evaluated by an experienced ophthalmologist (F.S.). Macular vessel density was analyzed at four levels: the superficial retinal capillary plexus (SCP), the deep retinal capillary plexus (DCP), the outer retinal layer (ORL), and the choroidal capillaris (CC) (^16^). It is acknowledged that differences in wavelength and light source can lead to variable detection of choroidal vasculature across devices (^17^). The density map was displayed in five regions (foveal, nasal, temporal, superior, and inferior), and the vessel density of each region was automatically calculated as a percentage. To minimize daily fluctuations, all scans were performed within a standardized window (13:00-14:00) for all participants. The primary outcome measures were smoking status, serum selenium level, and retinal vascular density measurements by OCTA.

### Statistical analysis

The statistical analyses were performed using the SPSS software (IBM SPSS Statistics for Windows, IBM Corp., v. 28.0, Armonk, NY) analyses. Mean, standard deviation, median, minimum and maximum values, frequencies, and percentages. Normality was assessed via the Kolmogorov-Smirnov test. Mann-Whitney U test was used to compare quantitative variables between groups. The Chi-square test was used for comparisons of qualitative variables. Statistical significance was set at *p* < 0.05.

## RESULTS

The mean age of smokers (female: n = 19; male: n = 5) was 39.7 ± 12.7 years; non-smokers (female: n = 12; male: n = 8) had a mean age of 42.3 ± 13.3 years; the control group (female: n = 18; male: n = 14) had a mean age of 37.3 ± 11.1 years. No statistically significant differences were observed between groups in terms of age or gender distribution (*p* > 0.05 for all). TAO patients who smoked had significantly higher CAS and TSI levels, and significantly lower selenium levels than non-smokers (*p* = 0.016, *p* = 0.027, and *p* = 0.042, respectively). There were no significant differences in thyroid-stimulating hormone, free triiodothyronine, and free thyroxine laboratory values between smokers and non-smokers in the TAO group (*p* = 0.521, *p* = 0.359, and *p* = 0.497, respectively) (**Table 1**).

**Table 1:** Demographic and clinical data of all subjects

Variables		Group			*p* ^*^	*p* ^**^	*p* ^***^
		Control (n = 32)	Smoker TAO (n = 24)	Non-smoker TAO (n = 20)			
Age (years) Mean ± SD (median)		37.3 ± 11.1 (1.5)	39.6 ± 12.1 (39.5)	42.3±13.3 (44.0)	0.872^m^	0.258^m^	0.453^m^
Sex, n (%)	Female	18 (56.3%)	19 (79.2%)	12 (60%)	0.073^X^2^^	0.790^X^2^^	0.165^X^2^^
	Male	14 (43.7%)	5 (20.8%)	8 (40%)			
Proptosis, mm		N/A	21.6 ± 3.3 (20.5)	21.7 ± 3.2 (22.0)			0.7410.936
Axial length, mm		23.1 ± 1.2 (23.0)	22.9 ± 1.1 (23.0)	23.1 ± 1.2 (23.0)			
CAS (range: 0-7)		N/A	1.0 ± 0.9 (1.0)	0.4 ± 0.7 (0.0)			0.016^m^
TSI (IU/L)		N/A	14.1 ± 16.1 (5.7)	4.88 ± 6.11 (3.2)			0.027^m^
Se (µg/L)		N/A	82.4 ± 21.9 (86.1)	102.1 ± 18.9 (102.7)			0.042^m^
TSH (µIU/mL)		N/A	1.3 ± 1.8 (0.6)	0.8 ± 1.0 (0.6)			0.521^m^
fT3 (pg/mL)		N/A	5.4 ± 5.3 (3.5)	3.8 ± 2.7 (3.3)			0.359^m^
fT4 (ng/dL)		N/A	2.0 ± 1.9 (1.4)	2.0 ± 2.3 (1.3)			0.497^m^

TAO: Thyroid-associated orbitopathy; N/A: not applicable; TSH: thyroid stimulating hormone; fT3: free triiodothyronine; fT4: free thyroxine; TSI: thyroid stimulating immune globulin; CAS: clinical activity score; Se: selenium.

X^2^: Chi-Square test, m: Mann-Whitney U test, *p* ≤ 0.05 indicates a significant difference.

*p: for control group vs smoker TAO group, **p: for control group vs non-smoker TAO group, ***p: for smoker TAO group vs non-smoker TAO group.

Central SCP and central DCP vascular densities were significantly lower in both smoking and non-smoking TAO patients than in the control group (*p* = 0.029, *p* = 0.017, *p* = 0.011, *p* = 0.004, respectively). No significant differences were observed in ORL-VD and CC-VD parameters by OCTA among groups (*p* > 0.05 for all). Among inactive TAO patients, OCTA-derived macular vessel density parameters did not differ significantly between smokers and non-smokers (*p* > 0.05 for all) (**Table 2**).

**Table 2:** Comparisons OCTA parameters between groups

OCTA parameters	Group																			*p* ^*^	*p* ^**^		*p* ^***^		
	Control (n = 32)			Smoker TAO (n = 24)							Non-Smoker TAO (n = 20)														
	Mean ± SD (median)			Mean ± SD (median)							Mean ± SD (median)														
VD of SCP (%)																									
Central	22.6 ± 3.1		22.9	21.2 ± 6.0					20.2		20.3 ± 3.3							20.3		**0.029^m^**	**0.017^m^**		0.872^m^		
Superior	48.7 ± 3.4		49.5	47.5 ± 4.2					48.5		48.3 ± 4.3							49.6		0.131^m^	0.802^m^		0.412^m^		
Nasal	46.8 ± 2.0		46.7	46.3 ± 2.4					46.9		46.1 ± 2.5							46.7		0.718^m^	0.429^m^		0.896^m^		
Inferior	47.9 ± 2.4		48.1	47.4 ± 3.9					47.2		46.1 ± 3.6							47.0		0.435^m^	0.561^m^		0.332^m^		
Temporal	47.9 ± 1.8		47.8	47.3 ± 2.2					47.6		47.6 ± 2.2							47.8		0.638^m^	0.803^m^		0.968^m^		
VD of DCP (%)																									
Central	20.1 ± 3.6		20.1	17.0 ± 6.3					15.4			16.1 ± 5.1					16.1			**0.011^m^**	**0.004^m^**		0.825^m^		
Superior	49.9 ± 3.3		50.3	49.0 ± 4.6					50.0			48.8 ± 5.0					49.1			0.357^m^	0.362^m^		0.849^m^		
Nasal	48.3 ± 1.9		48.6	47.4 ± 2.6					48.2			46.8 ± 2.7					47.4			0.183^m^	**0.013^m^**		0.352^m^		
Inferior	49.2 ± 2.4		49.5	48.4 ± 3.0					48.2			46.2 ± 5.9					47.7			0.322^m^	0.073^m^		0.332^m^		
Temporal	47.0 ± 2.5		47.2	46.5 ± 3.1					46.8			46.0 ± 3.5					45.4			0.617^m^	0.230^m^		0.459^m^		
VD of ORL (%)																									
Central	41.2 ± 3.5		41.2		42.7 ± 2.9					42.0			40.8 ± 3.2						41.3	0.118^m^	0.904^m^		0.204^m^		
Superior	48.9 ± 2.0		48.9		49.2 ± 2.0					49.5			50.1 ± 1.6						49.5	0.412^m^	0.076^m^		0.362^m^		
Nasal	49.0 ± 1.8		49.5		49.3 ± 1.3					49.4			50.5 ± 3.0						50.6	0.810^m^	0.062^m^		0.126^m^		
Inferior	48.9 ± 1.7		49.0		49.5 ± 2.0					49.9			50.0 ± 1.7						49.7	0.057^m^	0.085^m^		0.764^m^		
Temporal	48.8 ± 1.8		48.7		49.4 ± 2.2					48.9			50.3 ± 2.9						49.2	0.555^m^	0.149^m^		0.280^m^		
VD of CC (%)																									
Central	52.5 ± 2.0		52.2		52.6 ± 2.6					52.3			52.2 ± 2.6						52.2	0.960^m^	0.741^m^		0.904^m^		
Superior	53.0 ± 1.7		53.0		52.5 ± 2.3					51.9			52.5 ± 2.6						52.7	0.177^m^	0.561^m^		0.741^m^		
Nasal	52.5 ± 1.6		52.1		52.5 ± 2.0					52.7			52.5 ± 2.8						52.7	0.857^m^	0.936^m^		0.849^m^		
Inferior	52.9 ± 1.7		53.1		53.5 ± 1.6					53.3			52.4 ± 2.7						52.7	0.406^m^	0.429^m^		0.218^m^		
Temporal	52.7 ± 1.2		52.6		53.1 ± 2.2					53.5			52.2 ± 2.6						52.2	0.258^m^	0.976^m^		0.718^m^		

TAO: thyroid-associated orbitopathy; OCTA: optical coherence tomography angiography; VD: vessel density; SCP: superficial retinal capillary plexus; DCP: deep retinal capillary plexus; ORL: outer retinal layer; CC: choriocapillaris.

Data are presented asmean ± standard deviation, X^2^: for Chi-Square test, m: mann-Whitney U test, *p* ≤ 0.05 indicates a significant difference.

* *p:* for control group vs smoker TAO group,

** *p:* for control group vs non-smoker TAO group,

*** *p:* for smoker TAO group vs non-smoker TAO group.

## DISCUSSION

TAO is a debilitating autoimmune disorder of the orbit, predominantly affecting patients with Graves’ disease. Among various modifiable risk factors, smoking has been consistently identified as the most significant environmental contributor to TAO development, severity, and treatment resistance (^18^-^20^). The pathogenic mechanisms in TAO include oxidative stress, fibroblast activation, immune dysregulation, and increased production of inflammatory cytokines-processes exacerbated by smoking (^21^,^22^).

This study demonstrated that smoking is associated with significantly increased CAS and TSI levels, and significantly decreased serum selenium levels, in patients with inactive TAO. These results support prior reports suggesting that smoking alters selenium status and contributes to systemic oxidative imbalance (^23^,^24^). Our findings provide substantial evidence that smoking contributes to TAO pathogenesis and may prolong low-grade autoimmune activity during clinically inactive stages.

This study addresses a significant gap in the current literature. While previous studies have assessed retinal microvascular changes in TAO using OCTA, few have investigated such changes in the inactive phase, and none have simultaneously assessed serum selenium status, autoimmune markers (TSI), and smoking exposure. Some studies have reported vascular dropout in active TAO, while others have identified reduced macular perfusion in inactive TAO without considering modifiable systemic factors such as selenium or smoking (^25^-^27^). In one study comparing TAO patients who smoked and those who did not, Ceyhanoğlu and cols. (^12^) found no significant difference in macular vascular parameters compared to controls, but peripapillary vascular parameters were significantly lower only in TAO patients who smoked versus controls.

It is noteworthy that while both central superficial and deep capillary plexus vascular densities were reduced in both smoker and non-smoker TAO groups compared to healthy controls, no significant difference was observed between TAO subgroups. This suggests that chronic TAO-associated inflammation, independent of smoking effects, may lead to subclinical retinal microvascular impairment, consistent with previous OCTA studies (^25^,^26^). Previous work has shown that smoking reduces vascular density in deep retinal capillary plexus independently of systemic disease (^28^,^29^). In our study, the absence of a smoker control group limited the ability to evaluate the additive effects of smoking on retinal microvascular structure in TAO.

This study provides evidence that smoking status is associated with serum selenium levels, clinical severity, autoimmunity, and retinal microvascular changes in inactive TAO. This multidimensional perspective offers novel insights into the intersection of systemic oxidative and inflammatory factors with localized orbital pathology and establishes a new framework for risk stratification in apparently quiescent disease stages.

In this cohort, smoking was associated with lower serum selenium levels and increased immune activation in TAO patients. Selenium is a well-established antioxidant that modulates thyroid hormone metabolism and limits free radical-mediated tissue damage (^30^,^31^). Randomized controlled trials have shown that selenium supplementation in mild TAO yields improved clinical outcomes, delayed progression, and enhanced quality of life (^31^). However, there is limited literature evaluating this association in the context of smoking.

The authors propose a hypothesis of significant relevance: that smoking may exacerbate selenium deficiency, thereby increasing oxidative stress and prolonging immune activation in TAO. This hypothesis is reinforced by previous research linking smoking with reduced selenium bioavailability and elevated systemic oxidative stress markers (^22^-^24^). Our findings indicated an association between smoking and lower selenium levels in TAO, highlighting the further prospective studies to determine whether selenium monitoring or supplementation in smokers with TAO provides clinical benefit, especially given the narrow therapeutic range of selenium.

The findings of this study also have important clinical implications. Central SCP and DCP vascular densities were diminished in all TAO patients, regardless of smoking status, compared to controls. These results are consistent with reports of early macular and peripapillary vascular dropout in both active and inactive TAO (^25^-^27^). The results of this study indicate the presence of persistent subclinical microvascular changes, most likely attributable to long-term orbital inflammation, tissue remodeling, or ischemia associated with orbital volume expansion. In contrast to previous studies that focused exclusively on the active phase, our findings demonstrate that vascular impairments may persist into the inactive phase. This underscores the value of OCTA as a noninvasive biomarker for identifying early or residual orbital dysfunction.

It is important to acknowledge the limitations of this study. The OCTA device utilized provides a limited 6 × 6 mm scan area, which restricts the generalizability of microvascular observations to the entire retinal or orbital vasculature. Additionally, OCTA is unable to measure flow velocity or directionality, both of which are essential for a comprehensive understanding of retrobulbar hemodynamics. Variability among OCTA devices and segmentation algorithms may also negatively impact the reproducibility of results across centers. Each device relies on a unique algorithm to detect flow, and clinicians should be aware that these differences can influence imaging results and lead to discrepancies in follow-up assessments. To mitigate these issues, ensure consistency, and enable accurate comparisons of OCTA measurements, patients should be imaged with the same device and scan size whenever possible across visits.

Other limitations include the retrospective design, small sample size, absence of a smoker control group, and lack of selenium level data in the control group. The retrospective design and small sample size may have limited the statistical power to detect more subtle subgroup differences. These limitations prevent a clear assessment of the relationships among smoking status, serum selenium levels, clinical severity, autoimmunity, and retinal microvascular changes in TAO, and also limit the generalizability of our findings to all TAO patients. Despite these constraints, our results provide preliminary data supporting the need for future prospective and longitudinal studies. We believe that such studies, particularly with larger sample groups, will clarify the need for selenium supplementation in TAO smokers and elucidate whether the microvascular changes detected by OCTA are meaningfully related to clinical outcomes.

In conclusion, the OCTA findings revealed a reduction in macular microvascular density among patients with TAO, particularly within the SCP and DCP layers, regardless of smoking status. Therefore, OCTA may serve as a valuable adjunct tool for detecting persistent vascular changes in TAO patients and for guiding therapeutic interventions. Additionally, the study observed that, among patients with inactive TAO, smoking was associated with increased autoimmune activity (as evidenced by elevated CAS and TSI levels) and decreased serum selenium levels, which may indicate ongoing subclinical inflammation and heightened oxidative stress. These results support the assertion that smoking cessation is an essential component of TAO management, even during periods of disease inactivity. Although patients with TAO who smoke exhibited lower selenium levels, prospective studies with larger sample sizes are necessary to clarify the clinical significance of selenium monitoring and to determine whether these findings can be generalized to the broader TAO population.

## Data Availability

the datasets used and/or analyzed are available from the corresponding author upon request.
